# Account of a Fresh Supply of Vaccine Virus from the Cow

**Published:** 1838-10

**Authors:** J. B. Estlin

**Affiliations:** Bristol


### ACCOUNT OF A SUPPLY OF FRESH VACCINE VIRUS FROM THE COW.

BY J. B. ESTLIN, ESQ., OF BRISTOL.

THROUGH the kindness of an acquaintance residing in Gloucestershire, who remembered my wish to procure some fresh vaccine virus from the cow whenever an opportunity offered, a small quantity was sent to me on the 18th of August upon a piece of glass, said to have been taken from a cow, and also some taken from the hand of a person who had contracted sores by milking the diseased cows. I lost no time in investigating the source of this unexpected supply, and on the morning of the 21st ult. visited a farm-yard in which were about twenty-five cows, nearly all of which had been attacked with an eruption upon the teats during the four preceding weeks; the last of those thus affected had become so about a fortnight previous to my visit. Irregularly circular scabs existed upon the teats of many; in some the surfaces were raw from having been rubbed by recent milking: from none of the sores did any fluid exude, and most of them had been dressed with an ointment into the composition of which acetate of copper appeared to enter. The duration of the disease in the cows I ascertained to have been about a fortnight from the time that each had become affected. The hands of those who had been engaged in milking them presented sores in various stages; in one or two persons an eschar only remained, in others soreness still existed; in a boy of about thirteen years of age there was a large inflamed vesicle between the finger and thumb of the right hand, occupying all the space from the third joint of the finger to the second of the thumb: the skin was yellow at this part, as if a quantity of pus had been under it; such, however, was not the case, for from a small opening through the thickened and yellow cuticle the boy squeezed out a perfectly limpid fluid, with which I charged some glasses. This vesicle had made its appearance six days before, at which time he had felt ill. The others before mentioned, who had been engaged in milking (all of them females), reported that they had felt very ill before they discovered the "gathering" on their fingers; one represented herself as having been so much indisposed with headach, pain in the back and loins, and general weakness, as to be apprehensive she was going to have a serious fever: all described the sores as extremely painful, and as having occasioned enlargement and tenderness in the axillary glands. The course of the disease in them, as in the cows, was about a fortnight; and though it was known to them that the cows had something the matter with their teats, several days elapsed before the milkers suspected their malady to depend upon the disorder in the animals: never having witnessed the complaint before in the cows, they were ignorant of the nature of the existing disease. All the persons I have now spoken of had been vaccinated in infancy, one of them by Dr. Jenner. I next inspected three children who had been inoculated, by means of a needle, with lymph taken from one of those who had received the disease from the cows. One, a boy of fourteen years of age, who had gone through vaccination formerly, had four large circular mahogany-coloured scabs upon his arm, with the outer red line of an extensive areola still remaining. He was vaccinated about a fortnight before I saw him; the areola, I was informed, had appeared upon the eighth or ninth day, and he had been very unwell from the complaint. The next was a child who had been vaccinated from one of the milkers only three days before; scarcely any inflammation or prominence were observable about the punctures, and I have since learned that inoculation produced no effect. The third case was a little girl, Jane ———, about five years old, who had never had the cow-pox or small-pox. She had been vaccinated for the first time from the discharge taken from the hand of one of the milkers, during the activity of the complaint, on the 11th of August. I saw her, therefore, on the eleventh day of the disease. On one arm were three large, fine, prominent, circular vesicles, flattened in the centre, and with some areola; on the other arm was one vesicle, much larger, and less circular. I was informed that, for three or four days after she was vaccinated, it was difficult to decide if the infection had taken effect; and I have subsequently learned that the areola, which I saw on the eleventh day, continued to increase till the thirteenth day, and that the child had been "very poorly." From this little girl I took a supply of lymph which was quite limpid, and flowed very freely. . . .

On my return to Bristol I employed, as soon as was practicable, the lymph with which I was furnished.

The matter from the cow produced no effect, though tried on several children, nor did that from the boy's hand. Of those vaccinated with the lymph from Jane ———, two only out of many were infected. One of these patients had one well-formed vesicle, the other had two. In both, the disease was late in coming on; in one of them no redness appeared at the base of the vesicle till the tenth day, and the areola was not fully formed till the thirteenth day. In this case, however (Sarah Owen's), each vesicle was very perfect, rising abruptly from the arm, its upper part almost overhanging the base; its surface was much flattened, and it yielded freely limpid fluid when punctured before the areola appeared. On the thirteenth day the child's body and extremities were covered with a rash, in patches, much elevated from the skin, and she was constitutionally indisposed. On the fifteenth day the surface of the vesicle was becoming brown, and the areola, rash, and general indisposition had disappeared. From these two children many others were vaccinated, and now a second set has been inoculated from these last. In the majority of cases the vesicles have been inflamed round their base about the fourth or fifth day, and the areola has become extensive on the ninth. The areola usually continues for three or four days. In some cases it has been considerable on the eighth day. The vesicles are large, very well marked, and yield an abundant supply of clear lymph, and in every case there has been a good deal of constitutional disturbance. Some who have been vaccinated upon one arm with lymph taken on the eight day from the other arm, have exhibited in the second vaccination a small vesicle surrounded with a miniature areola, appearing and subsiding with that upon the opposite arm. *Med. Gaz.* Sept. 15, 1838.

